# Environmental Chemical Assessment in Clinical Practice: Unveiling the Elephant in the Room

**DOI:** 10.3390/ijerph13020181

**Published:** 2016-02-02

**Authors:** Nicole Bijlsma, Marc M. Cohen

**Affiliations:** School of Health Sciences, RMIT University, Bundoora, Victoria 3083, Australia; s9711185@student.rmit.edu.au

**Keywords:** environmental chemical assessment, exposome, clinical practice, toxicant, environmental medicine, personalized medicine, systems biology

## Abstract

A growing body of evidence suggests chemicals present in air, water, soil, food, building materials and household products are toxicants that contribute to the many chronic diseases typically seen in routine medical practice. Yet, despite calls from numerous organisations to provide clinicians with more training and awareness in environmental health, there are multiple barriers to the clinical assessment of toxic environmental exposures. Recent developments in the fields of systems biology, innovative breakthroughs in biomedical research encompassing the “-omics” fields, and advances in mobile sensing, peer-to-peer networks and big data, provide tools that future clinicians can use to assess environmental chemical exposures in their patients. There is also a need for concerted action at all levels, including actions by individual patients, clinicians, medical educators, regulators, government and non-government organisations, corporations and the wider civil society, to understand the “exposome” and minimise the extent of toxic exposures on current and future generations. Clinical environmental chemical risk assessment may provide a bridge between multiple disciplines that uses new technologies to herald in a new era in personalised medicine that unites clinicians, patients and civil society in the quest to understand and master the links between the environment and human health.

## 1. Introduction

Human exposure to environmental chemicals has increased exponentially over the past decades and a growing body of evidence suggests that chemicals present in air, water, soil, food, building materials and household products are toxicants that contribute to many of the chronic diseases typically seen in clinical practice. Yet, despite the call from numerous organisations for regulatory reform and an increase in training on environmental health for clinicians, environmental chemical assessment is generally overlooked in clinical practice and environmental chemicals can be considered as an elephant in the room that is largely ignored.

The failure of genome-wide association studies to explain the vast majority of chronic diseases now afflicting 50% of people of working age [1], together with emerging research exploring aberrations in the epigenome and “exposome” (the total exposures seen during the organism’s life) in the aetiology of chronic disease [2], has led to a paradigm shift in our understanding of chronic “non-communicable” disease [3]. Furthermore, the “epidemiological transition” from infectious diseases in developing countries to chronic diseases in developed countries, has led to a fundamental reconsideration of the health impact of environmental exposures [4].

Innovative breakthroughs in biomedical research and technology encompassing the emerging “-omics” fields (epigenomics, nutrigenomics, metabolomics, toxicogenomics), and advances in the field of classical toxicology, have further contributed to a new understanding of the relationship between chronic diseases and exposures to environmental chemicals across the lifespan. This new understanding validates what Hippocrates stated centuries ago; that one’s diet, lifestyle and environment, has profound consequences on health and wellbeing [5], and has wide reaching ramifications for the practice of medicine that provides clinicians with unique and important roles to play in identifying and preventing environmental chemical exposures.

## 2. The Rise of Chemical Production and Exposures

The number of chemicals in the world is essentially unknown, yet the world’s largest database on chemical information—the Chemical Abstracts Service (CAS) Registry^SM^ established in 1907, currently contains more than 100 million chemicals [6] with around 200,000 new chemicals being added each week [7]. While many of these chemicals are produced by natural processes, or are inadvertently produced as by-products of fossil fuel combustion or other industrial processes [8], the number of chemicals commercially produced has increased exponentially in parallel with increasing industrialization. Commercial chemical production has risen from 1 million tons in 1930 to 400 million tons in 2001 [9], and over the past few decades the global sale of chemicals has increased by a factor of 25 from U.S. $171 billion in 1970 to US$4.1 trillion in 2012 [10]. As of 2012, the number of industrial chemicals on the global market was estimated to be around 143,835 [10].

A number of large population biomonitoring studies have revealed widespread chemical exposures from the “womb to the tomb” with levels in humans and wildlife that are known to cause adverse health effects. Such studies include the National Health and Nutrition Examination Survey in the USA [11], DEMOCOPHES survey in Europe [12], German Environmental Surveys in Germany [13], Flemish Environment and Health Study in Belgium [14], Esteban cross sectional survey in France [15], Russian Federation [16] and the BIOAMBIENT ES in Spain [17] in addition to national birth cohort studies conducted in Denmark (Danish National Birth Cohort) [18], France (French Longitudinal Study of Children Survey) [19], Norway (Norwegian Mother and Child Cohort Study [20], and Spain (The Spanish Environment and Childhood Research Network) [21]. There are also ongoing epidemiological studies such as the Cross-Mediterranean Environment and Health Network project, which aim to demonstrate an integrated methodology for the interpretation of human biomonitoring data that will allow researchers to quantitatively assess the impact of chemical exposures on human health [16]. Despite these efforts, human toxicity data is lacking for most chemicals in widespread use, even when population-wide exposures are documented [22].

Disturbingly, many environmental chemicals are found in human breast milk and the placenta where they directly affect the foetus [23]. A landmark study conducted by the Environmental Working Group identified 287 chemicals in cord blood, raising the profile of the widespread exposures to everyday chemicals [24]. More recently, the Canadian “pre-polluted study” identified 137 chemicals in cord blood, 132 of which are reported to cause cancer and 133 that cause developmental and reproductive problems in mammals [25]. The brain of a foetus and infant is particularly vulnerable as the central nervous system is the dominant repository of foetal fat and many environmental toxicants are lipophilic. Consequently the health impact of chemical exposures is most evident in paediatric medicine where chronic disease has overtaken infectious diseases as the major burden of paediatric illness [26]. The obvious and extensive impact of environmental chemicals on children’s health, has contributed to paediatrics being the first medical discipline to identify chemical exposures as an important health issue, with the American Academy of Paediatrics establishing an environmental health committee in 1958 and publishing its first edition of *Paediatric Environmental Health* for clinicians in 1999 [27].

While chemical exposure is ubiquitous in the general population, the Environmental Justice Hypothesis suggests that exposures are unevenly distributed. This hypothesis, which emerged in the 1980s following the publication of several studies in the USA [28,29,30,31,32] suggests that environmental hazards are inequitably distributed according to class and race [33]. Yet, the strict bifurcation of communities into categories of Environmental Justice and Non-Environmental Justice is problematic [34], because much of the literature is based on comparisons of exposure and risk between different populations, rather than on the toxicological and biological impacts of those exposures [35]. Furthermore, while some minority groups and those with lower socioeconomic (SES) status are likely to bear a greater burden of environmental toxicants given their lifestyle, proximity to waste sites, industrial emissions and poorer quality ambient air, biomonitoring studies have identified toxicants in all individuals, the type and amount of which varies depending upon lifestyle factors and geographical variation. For example higher SES individuals have been found to have higher burdens of mercury, arsenic, caesium, thallium, perfluorinated compounds, certain types of phthalates and benzophenone-3 as a result of their lifestyle (fish consumption, dental history, homegrown vegies, cosmetic and sunscreen use) [36]. In contrast, lower SES individuals have been found to have higher levels of lead, cadmium, antimony, bisphenol-A and other types of phthalates, which may be partially mediated by smoking, occupation and diet [36].

## 3. Environmental Chemicals and the Origins of Chronic and Complex Disease

The dramatic rise in the number of commercially produced chemicals has resulted in exposure to industrial chemicals being ubiquitous in both developed and developing nations and an increasing disease burden that is not yet fully understood. The World Health Organisation estimates that 4.9 million deaths and 86 million Disability Adjusted Life Years were attributed to environmental chemicals in 2011 [10] and that approximately one-quarter of the global disease burden, and more than one-third of the burden among children under the age of 5 is due to modifiable environmental factors [37]. A recent review further estimated that the disease burden in the European Union associated with exposure to endocrine disrupting chemicals alone, cost $209 billion or 1.23% of Europe’s GDP [38].

Many of the chronic diseases that have substantially increased in prevalence over the past 40 years, appear to be related in part to developmental factors associated with nutritional imbalance and exposures to environmental chemicals [39]. For example the “developmental obesogen” hypothesis is used to explain why the prevalence of obesity among school age children between the early 1970s and late 1990s has doubled or trebled [40]. Whilst obesity prevalence has begun to plateau, a growing number of chemical obesogens such as organochlorine pesticides [41,42,43], bisphenol A [44], PCBs and phthalates [45] have been found in-utero and are implicated in the development of obesity later in life [46,47].

The concept of early life origins of disease was first brought to light in 1934 by Kermack and colleagues who suggested that decreased death rates due to all causes were the result of improved childhood living conditions [48]. This was later expanded upon by Neel in 1962 [49], Forsdahl in the 1970s [50,51], and in the late 1980s by David Barker who associated nutritional deficits during fetal development and consequent low birth weight, to increased risks for obesity, diabetes and cardiovascular disease and thereby came to be considered as the father of the “Fetal Origins of Adult Disease” hypothesis [52]. Whilst the Developmental Origins of Health and Disease (DOHaD) has historically focused on nutrition, understanding of the role of early life experience in chronic disease aetiology requires an integrated analysis of all aspects of the environment (nutrition, psychosocial stress, drugs, microbiome and environmental pollutants) and how they interact to cause disease [53]. Thus, the DOHaD has far reaching implications in clinical practice, and implies a need for clinicians to undertake an extensive paediatric, environmental and occupational exposure history and consider the role of nutrition and environmental chemical exposures during critical windows of development to understand the development of chronic illness in later life.

The list of diseases that may be caused or exacerbated by environmental chemical exposures is extensive and growing. These diseases include diabetes [54,55], infertility [56,57,58], testicular dysgenesis syndrome [59,60] which encompasses hypospadias [61,62], cryptorchidism [63,64], testicular cancer [65], and poor semen quality [66,67,68], ovarian dysgenesis syndrome [69], neurodegenerative diseases such as Alzheimer’s Disease [70], respiratory disorders such as asthma [71] and chronic obstructive airway disease [72], as well as autoimmune diseases [8], obesity [73,74,75] and cardiovascular disease [76,77,78]. Emerging evidence is also linking industrial chemicals to a pandemic of neurodevelopmental disorders [79] the implications of which have devastating consequences on family’s and the global economy [80,81]. Whilst the cause of these neurodevelopmental problems is not yet clear, genetic factors are acknowledged as only playing a minor role [82,83] and several hypotheses point to environmental influences involving aberrations in the gastrointestinal microbiota [83], industrial chemicals [81,84,85], malnutrition [86,87], viruses and drugs [88] as potential causal agents.

Environmental factors are also believed to account for a significant portion of cancer mortality worldwide [89]. There is a growing body of evidence associating various toxicants with cancer including: air pollutants like asbestos, radon, hexavalent chromium, tobacco smoke and benzo(a)pyrene with lung cancer [90,91,92,93]; endocrine disrupting chemicals such as pesticides, dioxins, furans and PCBs with an increased risk for breast cancer [94], endometrial, testicular and prostate cancer [95,96,97,98]; arsenic and disinfection by-products with bladder cancer [99,100]; vinyl chloride with liver cancer [101], benzene with leukemia [102]; and pesticides with childhood leukaemia [103,104,105]. Even though the incidence of cancer attributable to environmental chemical exposures has not been definitively established [106,107], the World Health Organization and the International Agency for Research on Cancer (IARC) suggest that between 7% and 19% of all cancers are attributable to toxic environmental exposures [108,109]. According to cancer biologists, this estimate is likely to be a gross underestimation, as many supposedly non-carcinogenic chemicals that are ubiquitous in the environment have been shown to exert low-dose effects that may contribute to carcinogenesis [110,111]. This is of particular concern in light of the fact that cancer has now become the world’s leading cause of mortality [112].

Clinicians are also seeing a rise in the prevalence of patients with a shopping list of ongoing seemingly unrelated persistent complaints, which some have described as a “pandemic of idiopathic multimorbidity” [113]. While multimorbidity is associated with chemical sensitivity, it presents an increasingly common and confusing primary care dilemma often labelled as Chronic Fatigue Syndrome [114,115], Systemic Exertion Intolerance Disease [114], Sensitivity-Related Illness [116], Idiopathic Environmental Intolerances [117], Fibromyalgia [118], Electromagnetic Hypersensitivity [119], Sick Building Syndrome [120] and Multiple Chemical Sensitivity [114]. These conditions are diagnoses based on exclusion rather than any specific aetiology as they have no clear aetiology, pathogenesis, or recognised genetic or metabolic markers that can be observed with standard laboratory testing. Despite the fact that the degree of hypersensitivity often parallels the intensity of the total body burden of bio-accumulated toxicants [121], patients with these conditions are relatively understudied [122] and are frequently considered to have psychogenic illness. Such patients have complex needs, and frequently present with a multitude of health complaints in different organ systems that often require attention from a range of medical specialists [123]. It has been suggested that a common aetiological pathway for a diverse range of idiopathic environmental intolerances may involve environmental chemicals inducing oxidative stress and subsequent mitochondrial dysfunction [124,125], in addition to low-grade systemic inflammation in multiple organ systems [124,126], and polymorphisms in nitric oxide synthase [125], antioxidant and/or detoxification genes [116,124], that result in a “toxicant-induced loss of tolerance” [127,128]. It is further suggested that exposures occurring during critical windows of development play an important role and that early life exposures are significant contributors to chronic diseases throughout the lifespan and across generations [96,129].

## 4. Chemical Risk and Chemical Risk Assessment

In contrast to the great majority of acute conditions and infectious diseases where cause and effect can easily be established, exposure to low levels of thousands of environmental chemicals over a life span requires a paradigm shift in the way in which causality is established. Chemical risk is based on the type and dose of chemical, combination effects, the timing of exposure, and individual risk factors, yet the existing chemical risk assessment framework only involves hazard identification and exposure assessment [130], where hazard identification assesses the ability of a chemical to cause harm at various dosage levels, and exposure assessment evaluates the dose that might be received at target tissue after contact. Such assessments rely heavily on data extrapolated from human epidemiology, animal testing and cell culture/*in vitro* laboratory studies [131] that fail to account for multiple routes of exposure, mixture effects, transgenerational epigenetic effects or individual human risk factors such as age, gender, genetics, nutrition, psychosocial determinants and comorbidities [130,132,133,134].

### 4.1. Dose Response and Low Dose Effects

Dose-response relationships follow the path laid by epidemiologist, Sir Austin Bradford Hill, and form the basis of most contemporary systems for chemical risk assessment and causation analysis [135]. Such assessments involve giving increasing levels of an individual chemical to a group of test animals with the key objective of providing a dose-response assessment that estimates a point of departure (traditionally the no-observed-adverse-effect (NOAEL) level or the lowest-observed-adverse-effect level), which is then used to extrapolate the quantity of substance above which adverse effects can be expected in humans [110]. Endocrine disrupting chemicals pose a particular dilemma for chemical risk assessment as these chemicals can exhibit non-monotonic dose-responses whereby the effect of low doses cannot be predicted by the effects observed at high doses [110,136]. In addition to the complexities involved with endocrine disruption, carcinogenesis is a highly complex process and a growing number of scientists are questioning the use of linear dose-response models for classifying carcinogens, as these models do not account for the complex and permutable pathogenesis of many cancers [137].

### 4.2. Chemical Mixtures and “Something from Nothing” Effects

The prediction of health risks based on NOAEL not only fails to account for non-monotonic dose-responses, it also fails to reflect real-life exposures which typically involves exposure to multiple chemicals [138]. This may explain why pesticide formulations such as “Roundup” have been shown to be significantly more toxic than their active principle (glyphosate), due to the inclusion of adjuvants that increase their potency yet are not accounted for in safety assessments [139]. Furthermore, the NOAEL approach does not consider “something from nothing” mixture toxicity whereby unpredictable additive, antagonistic or synergistic adverse effects may occur at doses around, or below points of departure [140]. For example, carpenters exposed to formaldehyde, terpenes and dust particles below their point of departure are reported to exhibit dyspnea, nose and throat irritation, chest tightness and productive cough [141] and complaints of headache, skin, eye, nose and throat irritation are reported in painters despite airborne exposure levels being below the known irritation levels for the single chemicals [142]. Similarly, weakly oestrogenic chemicals that are too small to be detected individually can jointly increase the actions of potent, endogenous sex steroids [143] and chemical mixtures can act synergistically to exert pro-carcinogenic and anti-carcinogenic effects that contribute to the accumulation of somatic mutations and instigate the hallmarks of cancer [110,144,145]. Inorganic arsenic is one such example. At high levels in drinking water, arsenic is a well-established human carcinogen associated with bladder, lung and skin cancer [146], however at lower doses, its cancer risk may depend upon other variables such as smoking, and on differences in individual susceptibility, either genetically based or via nutritional status or other conditions [147]. This observation parallels the well-established finding that smokers exposed to asbestos have a significant increase in lung cancer risk compared to non-smokers [148].

One theory of how chemical mixtures may elicit unexplained effects, is based on the observation that mixture effects commonly occur when chemical mixtures contain at least one lipophilic and one hydrophilic chemical [132]. Lipophilic chemicals promote the permeation of hydrophilic chemicals through mucous membranes [132]. This is important because lipophilic barriers in the body (skin and mucous membranes) serve as the body’s primary protection against the absorption of environmental chemicals [149]. The octanol-water partition coefficient, or K_ow_, which classifies the lipophilic character of a given chemical, is a useful parameter for environmental risk assessment that is used extensively by authorities in the European Union [150]. Most lipophilic toxicants can permeate the body’s membranes, and lipophilic chemicals with a K_ow_ greater than 2, are frequently used by the cosmetic industry as chemical penetration enhancers, as adjuvants in pesticides to increase the solubility of the active principle and by the pharmaceutical industry in drug-delivery systems to enhance transdermal drug delivery [151].

The evaluation of mixture effects is hampered by a lack of knowledge of the molecular pathways involved along with the large numbers of pollutants and their many potential combinations [152]. Lifetime effects of exposure to chemical combinations are also largely unstudied [111], and may only become evident after people have become sick [132]. Thus, until a risk assessment paradigm is designed for mixture effects, traditional risk assessment tools need to be used with caution when evaluating chemical mixtures [153].

### 4.3. Timing and Transgenerational Epigenetic Effects

Compelling epidemiological, pharmacological and toxicological evidence shows that there are several vulnerable periods of growth and development. During these periods, environmental interactions with the immune system and genome can increase susceptibility to central nervous system and metabolic diseases later in life [154]. Despite the fact that transgenerational effects arising from poor nutrition and chemical exposures in utero are widely reported in the scientific literature [155,156,157,158], the impact of epigenetic factors early in life remains largely unexplored in chemical risk assessment [159]. This is made more poignant by emerging evidence that in utero and early-life exposures may lead to disordered immune responses in adulthood and lead to heritable, epigenetic modifications in the immune responses of subsequent generations [137].

The first association of transgenerational inheritance of disease was documented in the Dutch famine of 1944 to 1945 where nutritional deprivation in utero was associated with increased risks for obesity later in life [160]. Epigenetic inheritance involving environmental chemicals is documented in the daughters of mothers who took the drug diethylstilbestrol (DES) to prevent miscarriages and later went on to have a significantly higher risk of vaginal cancer and other health complaints [161]. Similarly, emerging evidence of transgenerational effects in animal models links autism spectrum disorders to an array of environmental factors such as stress or environmental enrichment, endocrine disruptors such as vinclozolin and BPA, and inadequate nutrition [162].

Whilst the mechanisms by which the effects of exposure are transmitted through the germline to the next generation are still unclear, the most plausible explanation for these associations is the occurrence of epigenetic modifications involving DNA methylation, retained histone modification, tRNA fragments, and non-coding RNAs in somatic and germ cells arising from exposure to various environmental agents during critical windows of development [163]. Genome-wide association studies, in contrast to single nucleotide polymorphisms (SNPs) are likely to provide an important tool to identify the “susceptible biomarkers” to environmental chemicals [100]. The study of gene-environment interactions however, poses special challenges for clinicians because it requires the integration of complex information derived from a comprehensive exposure history, assessment of nutritional status and detoxifications pathways, and genetic profile.

### 4.4. Individual Factors

There are many individual factors that determine chemical exposure and risk of adverse health outcomes. They include; age, gender, ethnicity, genetics, nutritional status, intestinal microbiota and other lifestyle factors such as diet, smoking, exercise and hobbies, psychosocial determinants and comorbidities [130,132,133,134], the co- or pre-administration of other drugs, [132,164] and epigenetic states [165]. Exposure to toxicants also varies widely amongst individuals depending upon: past and current environmental exposures; occupation and health and safety practices; place of residence, work and/or school (proximity to vehicle exhaust, industry, mining, waste sites, industrial accidents, golf courses, parks, farms, flight paths, *etc*.) which is likely to be influenced in part by socioeconomic factors [36]; use of household products, chemicals and pesticides and appropriate use of safety equipment; and access to clean air, water, food and soil.

### 4.5. New Horizons in Chemical Risk Assessment

Advances in the “omics” fields such as genomics, proteomics and metabolomics, enable the screening of effects of chemical mixtures at the molecular level and the development of more sensitive and specific methodologies for biological monitoring of combined exposures [138,166]. High-resolution metabolomics (HRM) that uses ultra-high resolution mass spectrometry with minimal sample preparation can support high-throughput relative quantification of thousands of environmental, dietary and microbial chemicals and measure metabolites in most endogenous metabolic pathways, thereby providing simultaneous measurement of environmental exposures and their biologic responses [167]. Renewed interest in the placenta as a potential biomarker of exposure and its contributions to long-term human health and disease was recently initiated by the National Institutes of Health: Human Placental Project following evidence of its impact on the health of the mother [168,169] and fetus [170,171,172,173]. Prospective follow-up birth cohorts to examine the effects of early life programming will also be important [163]. Emerging technologies are also providing a mechanism to assess the allostatic load at the clinical level. For example, biomolecular adducts formed when a xenobiotic or its metabolite binds to biological molecules (DNA or proteins), are a useful tool to assess exposures to non-persistent chemicals in blood such as organophosphates and aromatic amines before clinical consequences appear [174].

Chemical risk assessment can be vastly improved by gaining better information about the totality of exposures across the life span (exposome) [175]. Prospective, population-based cohort studies have recently started to implement these methods using the exposome framework [166]. Consequently environmental health scientists are exploring new ways to strengthen the integrity of chemical risk assessment using the principles of systematic review [176,177] and some new initiatives are contributing to the refinement and codification of methodological approaches for systematic review and meta-analysis tailored to the specificities of environmental health [178]. To date there have been several attempts at establishing systematic reviews for evaluating data on chemical toxicity [179] including The Navigation Guide [176], the Evidence-Based Toxicology Collaboration [180], the PROMETHEUS project by the European Food Safety Authority [181], Integrated Risk Information System (IRIS) and Tox21 by the joint US EPA, Food and Drug Administration and National Institute of Environmental Health Sciences [182,183], ToxRTool by the European Commission [184], REACH by the European Chemicals Agency and KIimisch Ring Test [185].

Further developments to improve toxicity testing based on animals include a new design from the National Research Council for cellular-response networks that take into consideration advances in toxicogenomics, bioinformatics, systems biology, epigenetics, and computational toxicology thereby allowing scientists to uncover how environmental chemicals may lead to toxicity [134]. Emerging tools like the maximum cumulative ratio will further help to identify a person’s cumulative exposure to multiple chemicals over a lifetime [186].

## 5. The Challenges and Failure of Chemical Regulation

Once an industrial chemical has been tested and its point of departure has been established, it is up to government organisations such as the Environmental Protection Agency, US National Institute for Occupational Safety and Health, Safe Work Australia, European Commission’s Scientific Committee on Occupational Exposure Limit Values and non-governmental organisations like the American Conference of Governmental Industrial Hygienists (whose guidelines have been widely adopted in English speaking countries) to develop ambient air and occupational exposure limits. This process is frequently conducted in consultation with industry, involving scientists employed by various corporations, taking into account what is easily achievable in the workplace [187,188], along with a consideration of economic output and future innovations.

Legislation and the threat of litigation is a powerful motivating force that encourages employers and manufacturers of industrial chemicals to comply with their occupational health and safety requirements. Yet, exposure standards frequently differ from country to country depending upon the approach adopted. In the USA, the “low dose linear extrapolation” approach is favoured and legislated through the Toxic Substance Control Act (TSCA), as opposed to the “margin of exposure” approach used in Europe and regulated through REACH (Registration Evaluation, Authorisation and Restriction of Chemicals) [189]. The stark difference between the two is that REACH is a preventative approach that places the burden of proof on industry to show safety, as opposed to TSCA where the burden of proof is on the government to show harm [130]. As both of these systems primarily focus on industrial chemicals, numerous regulatory authorities have been established to regulate chemicals in food, cosmetics, pesticides and medicinal products. The chemical industry has exploited the inadequacies of weak laws and regulations, inefficient enforcement, regulatory complexity and fragmented overlapping authorities, to enable the introduction of untested chemicals into the commercial products and the environment [111,190]. Furthermore, non-occupational exposure standards for indoor air quality in residential environments are lacking, despite organisations such as the World Health Organisation [191] and the US Environmental Protection Agency [192] producing numerous reports and guidelines on indoor air quality.

The wide variation in exposure standards across jurisdictions along with vast numbers of commercial chemicals in widespread use that have not been adequately assessed for neurodevelopmental toxicity, endocrine disruption or other toxic effects, points to inadequacies in current chemical risk assessment procedures. Such inadequacies were highlighted as early as the 1970s by Bruce Ames who subsequently developed the Ames test for assessing the mutagenic potential of chemical compounds [193]. More recently, existing chemical risk assessment practices have come under scrutiny from various governmental and non-governmental bodies including the US Environmental Protection Agency [194], National Resource Defence Council [190], European Union (who responded by developing REACH), the National Academy of Sciences and the Institute of Medicine [130] and medical organisations such as the American Medical Association [195] and the American Academy of Paediatrics [196].

The availability of scientific information is fundamental to the ability to understand and manage risk and form the basis for regulatory action. However, while compelling epidemiological, animal and *in vitro* evidence is required to prove harm from a chemical exposure [111], there is a lack of well-accepted tools to objectively, efficiently and systematically assess the quality of published toxicological studies [197] making it difficult to assess health risks associated with low level exposure to hundreds of chemicals over a life time. Thus, for almost every conclusion about chemical-related health risks, it is possible to find a dissenting view [179] and the vast majority of scientific reviews conclude that “more research is needed”.

## 6. What Are the Barriers for Chemical Assessment in Clinical Practice?

While chemical risk assessment requires the application of multiple scientific fields to public health and regulatory issues, it is up to individual clinicians to determine the relevance of the many issues involved to the current and future health needs of their individual patients. Environmental chemical assessment in clinical practice therefore requires the personalization of medicine using the translation of a complex knowledge base to determine an individual’s body burden of toxicants, along with their personal risk factors and health status. Yet, despite the many scientific developments occurring in the area of chemical risk assessment, the discrepancies amongst leading authorities in their interpretation for evidence of harm makes it difficult for clinicians to translate scientific information into clinical practice. Furthermore, while clinicians must deal with the consequences of environmental chemical exposures and remain one of the most often accessed and most trusted sources of information about the health and the environment [198], there is widespread agreement that clinicians lack adequate information and training with respect to environmental risks and health [199,200].

### 6.1. What is Environmental Medicine?

A lack of clinical training in environmental medicine may be partly due to inconsistencies in defining “environmental medicine” and the confusion as to where it fits in relation to other specialties such as public health and occupational medicine. In the mainstream scientific literature, environmental medicine is defined as the evaluation, management, and study of detectable human disease or adverse health outcomes from exposure to external physical, chemical, and biologic factors in the general environment [199,201]. This is in contrast to occupational clinicians whose definition of environmental medicine varies depending upon the country and include: “exposures arising from industrial activities at a workplace” (Australia), “embracing any influences on health and disease that are not genetic” (UK), or as “a branch of medical science which addresses the impact of chemical or physical stressors on the individual or group in a community” (USA) [202]. To add to the confusion, public health clinicians define environmental medicine more broadly as “issues in the physical environment which impact on health, encompassing quality of air, water and food” [202]. Consequently environmental medicine has become a specialty field under the guise of “occupational and/or environmental medicine” and “public health” with the majority of “environmental physicians” focusing on public health issues rather than patient-centered clinical practice [203]. Wide inconsistencies in the definition for the term “environment”, has further ramifications for establishing environmentally attributable risk estimates. For example researchers and publications that define the environment in the narrow sense (air, water, food, and soil pollutants) tend to have smaller attributable risk estimates, whereas researchers and publications that refer to the environment in the broadest sense (including lifestyle factors, occupational exposures, and pollutants) have consistently larger risk estimates [106].

Relegating environmental health to a specialty field is highly problematic when environmental chemical exposures are implicated in many of the conditions seen by clinicians on a daily basis, yet the tools and expertise to adequately assess or manage these exposures are not widely available [200]. Furthermore, few doctors take adequate occupational or exposure histories [204] or refer patients to environmental physicians [123] and therefore environmental exposures are seldom identified in disease causation [111]. Consequently the cadre of environmental oncologists, researchers and clinicians trained in environmental health are relatively small, which may explain why environmental health is largely excluded from national policy [111].

### 6.2. Medical Training

There is a widely acknowledged need for greater awareness about chemical assessment amongst clinicians. The need for general clinicians to familiarise themselves with the health impacts of environmental chemical exposures was highlighted in 1967 at a conference by the American Medical Association’s National Congress on Environmental Health Management, and was further highlighted by the American College of Clinicians [205,206] as well as being a focus of the International Federation of Environmental Health in 1991 [207] and 2013 [208]. Despite the recognized need for clinical education on environmental chemicals, there appears to be a severe lack of environmental health education in medical undergraduate curricula. The Institute of Medicine has been particularly vocal about the lack of environmental health training as evidenced by the publication of the book “Role of the Primary Care Physician in Occupational and Environmental Medicine” [200], and the report “Environmental Medicine—Integrating a Missing Element into Medical Education” [199], which outlined a six competency-based learning objectives for medical students. This is further reinforced by the World Health Organisation’s report “Environmental Health and the Role of Medical Professionals” [209], which highlights the medical professionals role in assessing, investigating, diagnosing, monitoring, treating and preventing environmentally-related disorders.

The lack of environmental education for clinicians can be seen to be due to competition from other disciplines in crowded medical curricula, lack of funding and a lack of appropriately trained academics [210]. A significant proportion of undergraduate medical training is devoted to pharmacology as opposed to toxicology [211] or environmental health, with the exception of medical toxicology, a specialty field involving acute high dose exposures confined to emergency clinicians [212]. Furthermore, nutrition is rarely taught in undergraduate medical training despite the fact that the nutritional state of the person affects the impact and metabolism of toxicants in key Phase 1 and 2 metabolic detoxification pathways [132]. Not surprisingly, a recent survey of medical school graduates found that more than one-third of respondents said they received “inadequate” instruction in environmental health [213].

Both the World Health Organisation [214] and the American Academy of Paediatrics [27] acknowledge the lack of training in medical schools, and recommend that children’s environmental health be incorporated into the training for health care providers. Others have noted that obstetricians and gynaecologists are well positioned to prevent hazardous exposures in light of the irreversible impacts on health arising from chemical exposures in utero [215]. Aside from nutrition, smoking and drinking during pregnancy, obstetrics-gynecology education has been largely void of environmental health [216]. A recent report by the International Federation of Gynecology and Obstetrics recommended that reproductive and other health professionals advocate for policies to prevent exposure to toxic environmental chemicals, work to ensure a healthy food system for all, make environmental health part of health care, and champion environmental justice [85]. Calls to action are starting to be heard with the Association of American Medical Colleges recent webinar on “Teaching Population Health: Innovative Medical School Curricula on Environmental Health” [217] outlining the need to educate undergraduate medical students in environmental health which included links to the American College of Medical Toxicology’s Environmental Medicine Modules [218].

### 6.3. Environmental Health Data and Its Relevance to Clinical Practice

The sheer number of scientific journals, non-governmental organisations, associations, professional societies, environmental medicine practitioner organisations and governmental agencies dedicated to environmental health is enough to leave clinicians overwhelmed and despondent in ever gaining a grasp of this complex and ever growing field. The number of journals dedicated to public, environmental and occupational health under the category “Clinical Medicine” in the Web of Science database grew from 101 in 2005 to 142 in 2010 yet very little of the vast amount of literature on environmental health is actually published in general medical journals. This is likely to be due to a variety of factors. Firstly, evidence about environmental exposures based on animal studies in the absence of human experimental data is considered “weak evidence” by the medical fraternity and outside the comfort zone and time constraints of most clinicians [219]. In addition, whilst epidemiological studies are important tools for determining risk, they can be limited by often failing to take into account the role of individual differences reflected in subpopulations [220]. For much of the history of clinical trials, the treatments under investigation were assumed to apply to anyone with the relevant clinically defined condition [221]. However the emerging “omics” fields and molecular cancer epidemiology, has led to the recognition that clinical trials need to be redesigned to account for individual variations (*N* = 1) arising from one’s genomic profile, lifestyle and environmental exposures.

How do we accommodate evidence in the context of individual patients? As it is not viable to devote resources to generate randomised clinical data on patients whose variants are so unique that they represent a small minority of the community, a shift is needed in the way we think about evidence-based medicine. Despite rapid advances in technology and the volume of literature published about the adverse health effects arising from exposure to environmental chemicals, health care systems have fallen far short in their ability to translate knowledge into practice and to apply new technology safely and appropriately [222].

## 7. How Can Clinicians Assess Environmental Chemical Exposures?

Whilst biomonitoring is an established approach to evaluate the internal body burden of environmental exposures, the use of biomonitoring for exposome research is limited by the high costs associated with quantification of individual chemicals [167]. Interpretation of the presence of chemicals in human tissues has also been the subject of much controversy, as its presence cannot be taken to imply that there will be adverse functional consequences [123,223]. For example blood and urine samples generally only reflect recent exposures to toxicants (heavy metals, persistent organic chemicals, organophosphate (OPs) and carbamate pesticides); hair and nails reflect past exposures (pesticides, heavy metals, polychlorinated biphenyls and polyaromatic hydrocarbons), are easily contaminated and difficult to collect in a standardized way; and many other biological matrices such as human milk, saliva, adipose tissue and meconium lack reliable reference values for human populations [138].

Resources and tools to educate clinicians and elicit personal environmental health data in the clinical setting are limited in scope and applicability. For example, the Australian NHMRC 2011 Standard for Clinical Practice Guidelines portal does not provide any guidelines on how to assess environmental chemical exposures, despite the fact there are extensive guidelines for conditions like diabetes, which are known to be influenced by chemical exposures. The lack of guidelines is compounded by a lack of conventional pathology tests to assess environmental chemical exposures. In addition, the knowledge required to understand what, how and when to assess environmental chemical exposures requires extensive knowledge on individual toxicants, their metabolites and/or the product of toxicant interactions with endogenous targets [174], which is not generally considered within the realm of most clinicians.

Tools that are available such as the EPA’s Office of Pesticide Protection questionnaire, the CDC’s Agency for Toxic Substance and Disease Registry “Taking an Exposure History Guide”, The Navigation Guide, Eco-Health Footprint Guide (Global Health and Safety Initiative), Quick Environmental Exposure and Sensitivity Inventory (QEESI), WHO Paediatric Environmental History to name but a few, are unlikely to be known by most general clinicians, and have either not been validated and/or require lengthy periods of time to complete, which may not always be practical in a clinical setting. Consequently clinicians and those specialising in idiopathic multimorbidity, are likely to have developed their own assessment procedures to assess their patient’s susceptibility or exposure to environmental toxicants. Such procedures may include extensive historical inquiries (paediatric, occupational and environmental exposure histories) along with an assessment of their patients’ metabolic, nutritional, genetic, and exposure profiles and include unconventional tests performed at pathology laboratories from around the globe such as Acumen Labs, Nutripath, Doctors Data, Genova Diagnostics, Healthscope Functional Pathology, Mycometrics, US Biotek Laboratories, Great Plains Laboratory and AsureQuality Labs, amongst others. Such assessments may come at considerable expense to their patients and as exposure standards are not available for many biomarkers, these clinicians must interpret the data without the benefit of published normal ranges or specific diagnostic criteria.

To assess environmental chemical exposures in patients, the challenge for clinicians is to ask relevant questions to elicit toxicant sources and exposure, identify the most relevant tests and to digest data from multiple streams (traditional medical data, “omics” data and quantified self-data), and place this information in the context of individual patients in a way that has measurable and meaningful outcomes. Only by doing so, can medicine shift the focus from treating disease, to prevention and wellness.

### 7.1. Lessons in History—Asking the Right Questions

The history of medical care is littered with examples of missed opportunities, wasted resources and counter-productive policies, due to the inability to effectively assemble and act on available evidence on toxicant exposure [179]. Environmental tobacco smoke, asbestos, lead dust, benzene, polychlorinated biphenyls, chlorofluorocarbons, lead and organochlorine pesticides are just some examples where warnings were ignored decades prior to the emergence of devastating public health issues [224]. History also provides examples of doctors whose observations at the clinical level, in addition to the power of rapid action, resulted in significant improvements in public health. For example, in the 18th century, British surgeon, Sir Percivall Pott, without knowing the cause or mechanism of action, stopped an epidemic of scrotal cancer in chimney sweeps by asking them to improve their genital hygiene [225]. Furthermore, in 1854, Dr. John Snow who is credited as the first epidemiologist, was able to prevent an outbreak of cholera by dismantling a water pump handle in Broad St, London, despite great criticism from his peers [226].

### 7.2. The “Omics” Revolution and Personalised Medicine: A Match Made in Heaven

Following completion of the Human Genome Project in 2003 in conjunction with the rapid advances in bioinformatics, the “omics” fields exploded onto the scene, challenging our understanding of the nature and cause of disease, whilst also shifting the focus to what it means to be well. Clinical genetic testing has transformed from being centred on mutation detection for Mendelian disorders like sickle cell disease to personal genomic data as a way to predict ancestry and assess disease risk. The emergence of hand-held devices such as the “SNIP doctor” for analysing single nucleotide polymorphisms, has bridged the gap from the bench to the bedside [227]. Whilst the brunt of these discoveries has yet to infiltrate clinical practice (because it takes an average of 17 years to incorporate scientific discovery into clinical practice [222]), the ramifications of these findings will provide more precise treatment for individuals and issue a new era in personalised medicine.

Breast cancer risk provides a good example. Whilst the aetiology of breast cancer is still not fully understood, there are several known risk factors including: the age of menarche/parity/menopause; family history of breast cancer; length of time of breast feeding; body mass index; drugs (hormone replacement therapy, oral contraceptive pill); exercise; alcohol intake; and cigarette smoking [228,229]. Given that the prevalence of gene mutations (BRCA1, BRCA2) for women diagnosed with breast cancer are low (5.3% and 3.6% respectively) [230], it has been suggested that low-penetrance susceptibility genes combined with environmental factors may be important risk factors [231]. Advances in genomics have identified several gene variants (single nucleotide polymorphisms (SNPs)) in key detoxification pathways that maybe associated with breast cancer susceptibility [232,233,234,235,236]. However few of these variants (COMT, CYP1B1, GSTP1, MnSOD, MTHFR) have been shown to contribute to breast cancer risk individually except when these polymorphisms are combined [237], or in the presence of relevant environmental chemical and lifestyle exposures [238,239]. This is significant in light of the fact that unique populations of various ethnicity have been shown to have polymorphic variants in detoxification enzymes, which may predispose them to increased adverse health effects from environmental chemical exposures [240,241]. For example, despite the low incidence of breast cancer amongst Asian women [242], a recent meta-analysis to determine the role of MTHFR C677T polymorphism in breast cancer risk, showed a strong significant association between TT genotype and breast cancer which is far more prevalent in the Asian population compared with the Caucasian population [235]. This may explain why US-born Asian women have an almost two fold higher incidence of invasive breast cancer than foreign-born Asian women [243], implying that epigenetic effects involving lifestyle, dietary, and/or environmental factors are likely to play a role. The results of these findings, may explain why so many risk factors have been implicated in breast cancer and other chronic diseases, and yet a causal relationship has not been definitively established.

As stated by Dr. Francis Collins, Director of the US National Institutes of Health “*genetics loads the gun and the environment pulls the trigger*”. Thus establishing an individual’s risk to environmental chemicals based on the presence of low penetrance genes (SNPs) alone is limited unless it is combined with the potential epigenetic effects of pathological, developmental, dietary and environmental chemical exposure history across the lifespan [244]. The concept that the phenotype is the consequence of gene-environment interaction was highlighted by Archibald Garrod in 1902 who suggested that individual differences in genetics could play a role in variation in response to drugs, and that this effect could be further modified by the diet [245]. However, while genetic testing is providing greater understanding of disease risk, the application of gene testing at the clinical level is fraught with challenges.

Very few of the one million plus SNPs identified in genome wide association studies have clear functional implications and actionable outcomes that are relevant to mechanisms of disease [246], which is why clinicians perceive the analysis of genetic data as requiring considerably more time and work with uncertain outcomes [247]. Secondly clinical guidelines for genomic testing is still in its infancy, such that there is a poor understanding of the effect of individual alleles, many of which appear to be non-sense mutations but may at a later date prove to be of clinical relevance especially in the context of other alleles, epialleles and environmental exposures [248]. Furthermore, the accuracy of laboratory analysis of genetic information and interpretation of results may vary amongst direct-to-consumer genetic testing companies depending upon their quality control standards [249]. Despite the remarkable advances in biomedical research and in particular, the field of genomics in the past twenty years, concerns have been raised about the lack of knowledge and skills in genetic and genomic testing, interpretation of test results, communication of results to patients and families, and basic genetic counseling amongst general non-academic clinicians [250,251]. Finally and perhaps most importantly, clinical genomics requires an understanding of the ethical, legal and social considerations associated with genomic profiling including employment and health insurance nondiscrimination, patient’s rights, informed consent, disclosure, microarray screening for pregnancy, cost/benefit ratio, drawbacks *versus* perceived benefits, genetic counselling, protection of privacy and data protection [252,253,254]. Clinicians will therefore need educational programs that target relevant scientific, clinical, ethical, legal, and social topics and support systems that address structural and systemic barriers to the integration of genetic medicine into clinical practice [251].

### 7.3. Citizen Science and Mobile Technologies

It is clear that the impact of environmental chemical exposures is an issue that requires action at many levels and must ultimately include the general community. Thus, civil society, including non-government organisations and civilian advocates can play a vital role in shedding light on the nature and extent of chemical exposures and their impacts. As such, citizen science or “participatory urbanism” is an emerging field that shows great promise in the scope of environmental awareness and regulation [255]. This became evident as early as the 1960s when a citizen science project revealed widespread contamination from radioactive fallout from atomic weapon testing through the analysis of strontium 90 in baby teeth collected from around the world, leading to the signing of the Partial Nuclear Test Ban Treaty in 1963 [256].

The potential for participatory citizen science has expanded enormously since the 1960s. Consumer’s appetite for health information is evident by the 40,000+ smartphone health applications now available [257] and the fact that almost 60% of mobile phone users have downloaded a health app [258]. Furthermore, genomic profiling is now available for as little as $99 from companies like 23andMe who have databases in excess of one million clients. With more tools at their disposal, web applications have enabled citizens to take a proactive approach to make informed health-care decisions. No longer passive recipients of health care, these “e-patients” have given birth to the quantified self-movement, and irrevocably changed the traditional doctor-patient relationship making way for the participatory medical model. Furthermore, the advent of the internet along with rapid advances in mobile computing, wearable devices, nano-biosensors, lab on a chip technology, geographical information systems, the quantified-self movement, the internet of things, big data analytics and cloud computing, represent disruptive innovations that promise to create a fundamental shift in biological discovery. Such advances, which enable the real-time measurement of physiological and psychological states along with environmental measures, offer the ability to better predict, detect and prevent disease brought on by chemical exposures and thus radically accelerate our understanding of the health impact of environmental chemical exposures [259].

Widespread adoption of information technology applications requires behavioral adaptations on the part of clinicians, organizations, and patients [222] and the ability of technology designers to build better tools and platforms that allow patients to share data with their doctors in order to augment existing medical knowledge and practices [247]. Whilst citizen science has the potential to build important bridges between scientists, clinicians and the public with positive outcomes for all [260], clinicians need to be receptive to the shift in the availability of knowledge to the public and be capable of answering questions that might arise so they can direct patients to credible and reliable resources when appropriate [261]. Engaging volunteers in rigorous science, global-scale citizen science projects also provide an excellent opportunity to promote awareness, and educate and empower individuals and clinicians to find solutions to problems that would otherwise be large and overwhelming [260].

While citizen science and mobile technology has the potential to engage the wider community in monitoring and reducing exposures to environmental pollutants, currently there is a lack of integration between data sources and a key challenge is the integration of big health data streams. Incorporating “big data” arising from traditional medical data, “omics” data and quantified self-data to routine clinical care will be a formidable and challenging task, and yet one that is vital for the emergence of personalised medicine that is predictive, personalised, preventative and participatory (4Ps). This challenge has been taken up by the field of systems biology, which uses computational mathematical tools that promise to unify multiple data sets—personal, clinical, genomic, geographical and environmental data. Systems biology therefore provides the foundation for personalised medicine where the patient becomes an integral part of the identification and modification of disease related risk factors and the clinical decision-making processes takes advantage of the most up-to-date scientific knowledge [262].

### 7.4. Tomorrow’s Doctor

The failure of regulatory authorities to manage risk associated with environmental chemicals, in addition to the widespread and growing number of chemicals found in the human population, provides clinicians with unique and important roles to play in identifying and preventing environmental chemical exposures. While there some clinicians who are supported by integrative/functional medicine associations that are rising to meet this challenge, there are no standard practice models and these clinicians have had to engage in continual education, develop their own clinical assessment tools, and navigate a path through the complex landscape of laboratory tests and the emerging science in multiple fields. This is an extremely challenging task, as conducting environmental chemical assessment at the clinical level includes (but is not limited to):

- Establishing the patient’s inherent susceptibility to environmental chemicals through assessment of their demographics, ethnicity, socioeconomic status, comorbidities, nutritional and genomic profile.
- A detailed place history that includes places of residence and work across the lifespan and throughout the week including primary modes of transportation and an assessment of the patients living conditions including their proximity to traffic and other sources of air pollution and potential sources of lead and other heavy metals, mould, dust, indoor air pollution and chemicals in building materials, furnishing and consumer products.
- An obstetric, paediatric, environmental and occupational exposure history that includes a detailed dietary history, drinking water sources, pharmaceutical and recreational drug use and general lifestyle factors including the use of chemicals in the home and garden, cooking utensils, cleaning methods, personal care products and consumer goods.
- A family history that includes previous generations.
- A detailed symptom history that includes a timeline from the perinatal period and enquiry into multiple organ systems.
- A physical examination to look for physical signs of metabolic, neurological, reproductive or other disease and co-morbidities.
- Assessing current toxic load through performing various biomonitoring tests that include assessment of biomarkers in various body tissues to assess long term accumulation of toxicants as well as short term exposures.
- A consideration of external data sources such as geographical information systems and governmental or non-government environmental pollution reporting, ambient air monitoring, drinking water quality and any crowd-sourced data.
- Networking with other professionals who can assess the patient’s home and/or workplace to establish sources of exposure.
- Keeping up with the latest regulations and scientific information on environmental chemicals and how they may be assessed as well as their interactions with each other, different diseases and individual patient factors.

To achieve this, clinicians need to possess a cluster of related knowledge, skills and attitudes in the fields of genomics, nutrigenomics, microbiology, hygiene, toxicology, occupational health, public health, epidemiology and, from a clinical perspective, nearly all fields, as well as general medicine, paediatrics and oncology. In addition to clinicians dedicating themselves to this task, the politics and economics of contemporary medicine need to support the dissemination and implementation of this kind of information. However there are many obstacles that hinder this process including the complexities involved in integrating data from numerous emerging fields, time constraints imposed on clinicians, educational requirements, the need for population and individual biomonitoring, the lack of clinical assessment tools, pathology facilities and adequate risk based regulation, profit based funding models that favor treatment over prevention, and the lack of political will to implement drastic changes in how we produce, monitor and regulate chemicals. Ultimately the issues raised by environmental chemical exposures are far greater than those that can be faced by clinicians, as they affect all people and indeed all life on earth. There is a need therefore for concerted action at all levels including actions by individual patients, clinicians, medical educators, regulators, government and non-government organisations, corporations and the wider civil society in order to understand and minimise the extent of toxic exposures on current and future generations.

## 8. Conclusions

Large population biomonitoring studies have revealed widespread exposures to environmental chemicals at levels in humans known to cause adverse health effects. Despite emerging evidence associating many of these chemicals with chronic diseases typically seen in general clinical practice, environmental chemical assessment has largely been overlooked in clinical practice. Part of the reason lies in the scientific complexities involved, inadequacies in chemical regulations and chemical risk assessment, inconsistencies in defining environmental medicine, a lack of information on environmental chemicals in general medical journals, inadequate pre- and post-graduate medical education on environmental medicine, the limitations of current biomarkers and laboratory tests, along with time, funding and political constraints that limit the use of available tests in clinical practice.

Assessing environmental chemicals in clinical practice may very well be the toughest challenge facing medicine today. Determining susceptibility to environmental chemicals requires a sophisticated understanding of each individual patient and the use of increasingly refined approaches that incorporate extensive paediatric, environmental, geographical, occupational and lifestyle data. The clinical assessment of environmental chemicals also involves embracing the gene-environment paradigm and moving beyond reactive disease models to one that is proactive and preventative, whilst also acknowledging the vital role patients play in their own wellbeing. Recent developments in the fields of systems biology, and the “-omics” fields and advances in peer-to-peer wireless sensor networks, may soon offer tools that provide a bridge between multiple disciplines and herald a new era in personalised medicine that unites clinicians, patients and civil society in the quest to understand and master the links between the environment and human health.
